# NK Cell Activity Differs between Patients with Localized and Diffuse Cutaneous Leishmaniasis Infected with *Leishmania mexicana:* A Comparative Study of TLRs and Cytokines

**DOI:** 10.1371/journal.pone.0112410

**Published:** 2014-11-14

**Authors:** Isabel Cristina Cañeda-Guzmán, Norma Salaiza-Suazo, Edith A. Fernández-Figueroa, Georgina Carrada-Figueroa, Magdalena Aguirre-García, Ingeborg Becker

**Affiliations:** 1 Unidad de Investigación en Medicina Experimental, Facultad de Medicina, Universidad Nacional Autónoma de México, Hospital General de México, México, D.F., México; 2 Universidad Autónoma Juárez de Tabasco, Villahermosa, Tabasco, México; INRS - Institut Armand Frappier, Canada

## Abstract

*Leishmania mexicana* causes localized (LCL) or diffuse cutaneous leishmaniasis (DCL). The cause of dissemination in DCL remains unknown, yet NK cells possibly play a role in activating leishmanicidal mechanisms during innate and adaptive immune responses. We had previously shown that *Leishmania* lipophosphoglycan (LPG) is a ligand for TLR2, activating human NK cells. We have now analyzed NK cells in LCL and DCL patients. NK numbers and effector mechanisms differed drastically between both groups of patients: DCL patients showed reduced NK cell numbers; diminished IFN-γ and TNF-α production; and lower TLR2, TLR1, and TLR6 expression as compared to LCL patients. The altered protein expression found in NK cells of DCL patients correlated with their down-regulation of IFN-γ gene expression in LPG-stimulated and non-stimulated cells as compared to LCL patients. NK cell response was further analyzed according to gender, age, and disease evolution in LCL patients showing that female patients produced higher IFN-γ levels throughout the disease progression, whereas TLR2 expression diminished in both genders with prolonged disease evolution and age. We furthermore show the activation pathway of LPG binding to TLR2 and demonstrated that TLR2 forms immunocomplexes with TLR1 and TLR6. In addition to the reduced NK cell numbers in peripheral blood, DCL patients also showed reduced NK cell numbers in the lesions. They were randomly scattered within the lesions, showing diminished cytokine production, which contrasts with those of LCL lesions, where NK cells produced IFN-γ and TNF-α and were found within organized granulomas. We conclude that in DCL patients the reduced NK-cell numbers and their diminished activity, evidenced by low TLR expression and low cytokine production, are possibly involved in the severity of the disease. Our results provide new information on the contribution of NK cells in *Leishmania* infections of the human host.

## Introduction

*Leishmania mexicana* causes a wide spectrum of cutaneous diseases, ranging from localized cutaneous leishmaniasis (LCL), characterized by ulcers at sites of parasite inoculation, to diffuse cutaneous leishmaniasis (DCL), where parasites spread throughout the skin forming disfiguring nodules [1]. In Mexico, 400 new patients with cutaneous leishmaniasis are diagnosed each year, where the prevalence of DCL is less than 1% [2]. Although the cause for the uncontrolled parasite spread in DCL patients remains unknown, the early innate immune response against *Leishmania* possibly plays a pivotal role in determining disease evolution. *Leishmania* lipophosphoglycan (LPG) is a major surface molecule that activates TLR2 in cells of the innate immunity [3], [4]. The carbohydrate composition of LPG characterizes different *Leishmania* species [5], [6]. Murine models of leishmaniasis have linked various TLRs (TLR2, TLR3, TLR4 and TLR9) with enhanced IFN-γ and IL-12 production and parasite control [4], [7]–[10]. Among the first innate cells capable of early IFN-γ and TNF-α production are NK cells [11]. They can be divided into 2 subsets: CD56^dim^ and CD56^bright^, yet the roles of these subsets have not been clearly characterized in leishmaniasis [12], [13]. We had previously shown that *Leishmania* LPG activates human NK cells through TLR2 stimulation, leading to IFN-γ and TNF-α production [3]. These cytokines synergize in the macrophage to induce iNOS leading to NO production, one of the molecules responsible for intracellular *Leishmania* destruction [14]. Even though NK cells have been shown to play an important protective role in mouse *Leishmania* infections [11], [15], their response has not been analyzed in patients with LCL and DCL.

In the present study, we comparatively analyzed NK-cell activity as well as their response towards the parasite in LCL and DCL patients. We found that peripheral blood and lesional NK cells of DCL patients were severely reduced in number and produced markedly less IFN-γ and TNF-α, as compared to LCL patients. In addition to the reduced cytokine production, NK cells of DCL patients also showed diminished TLR2, TLR1 and TLR6 expression, both in LPG-stimulated and non-stimulated NK cells, which contrasted sharply with the heightened response found in LCL patients. The reduced NK cell cytokine production correlated with a down-regulation of IFN-γ gene expression in DCL patients. We further show the activation pathway of TLR2 by *Leishmania* LPG, and the participation of TLR1 and TLR6 in the binding of LPG.

## Materials and Methods

### Ethics Statement

The study was reviewed and approved by the Ethics and Research Committees of the Faculty of Medicine of UNAM (Universidad Nacional Autónoma de México) (FMED/CI/RGG/013/01/2008) and guidelines established by the Mexican Health Authorities were strictly followed. All patients and controls were informed and signed a written consent to participate in the study.

### Patients and controls

28 patients with LCL and 6 with DCL from La Chontalpa (Tabasco State), an endemic area in southeastern Mexico, were analyzed. Patients were diagnosed by clinical criteria, parasite demonstration in Giemsa-stained smears taken from lesions and intradermal Montenegro hypersensitivity test. LCL patients showed skin ulcers with few parasites, all were positive to the Montenegro test. DCL patients had multiple non-ulcerative nodules covering large areas of the skin that contained heavily parasitized macrophages. All DCL patients were negative for the Montenegro skin test. All patients received anti-*Leishmania* treatment with Glucantime.

Blood samples were taken from 28 LCL patients (17 males and 11 females), which had a mean age of 28 years and a disease duration ranging from 1.5 to 18 months. The LCL patients were divided according to gender, age (≤25 years and ≤26 years) and evolution time (≤3 months and ≥4 months). The 6 DCL patients (five males and one female) had a mean age of 44 years (24–57 years) with an average disease evolution of 18 years (two patients had 3 years, three had 17 years and one had 35 years). Blood from 21 healthy donors was obtained in a blood bank.

### Parasite culture

*Leishmania major* (MHOM/SU/73/5-ASKH) promastigotes were cultured in RPMI-1640 medium (Gibco, Grand Island, NY, USA) with 10% heat-inactivated FBS (Gibco) and 293 µg/mL L-glutamine (Sigma, St. Louis, MO, USA). Parasite infectivity was maintained through regular passages in BALB/c mice.

### Lipophosphoglycan purification

LPG was purified as previously described [3]. Briefly, parasites were sub-cultured every 4–5 days and grown to a density of 2×10^7^/mL. Promastigotes were harvested from stationary-phase cultures and centrifuged at 3200× *g* for 10 min and washed in PBS. The pellet was extracted with chloroform/methanol/water (4∶8∶3, v/v) during 30 min at RT. The insoluble material was used for LPG extraction with 9% 1-butanol in water (2×50 mL) and the pooled supernatants were vacuum dried. LPG was purified from this fraction by HPLC, using an octyl-sepharose column and a 1-propanol gradient (5–60%) in 0.1 M ammonium acetate. Two octyl-sepharose columns were used to optimize LPG purity. The preparations tested negative for endotoxin with the *Limulus* sp. amebocyte lysate assay (E-Toxate Kit; Sigma). Additionally, a sample of LPG was analyzed by SDS-PAGE to verify the absence of protein contaminants. 10 µg/mL LPG was used in all experiments.

### NK cell purification

NK cells were purified from PBMC of LCL and DCL patients, as well as from healthy donors. Briefly, PBMC were separated by density gradient (Histopaque-1077, Sigma-Aldrich) at 300× *g* for 20 min at 20°C. Cells were obtained from the interface, washed twice in cold PBS and placed in RPMI-1640 (Gibco), supplemented with 10% heat-inactivated FBS, 2 mM L-glutamine, 10 nM HEPES, 100 µg/mL penicillin-streptomycin (Gibco), 17 mM NaHCO_3_ and seeded in Petri dishes at 37°C, 5% CO_2_ during 18 h for adherence of monocytes. Non-adherent cells were removed, washed in PBS and NK cells were purified with an NK cell isolation kit II (Miltenyi Biotec, Bergisch Gladbach, Germany). Briefly, 1×10^7^ total cells was suspended in 40 µL PBS containing 10 µL of cocktail of biotin-conjugated monoclonal antibodies against CD3, CD4, CD14, CD15, CD19, CD36, CD123 and glycophorin A and incubated for 10 min at 4°C. 30 µL PBS and 20 µL anti-biotin microbeads were added for 15 min at 4°C. The cells were washed with PBS, centrifuged at 300×*g* for 10 min and passed through a magnetic separation LS column (Miltenyi). NK cells were isolated by negative selection. The purity of the enriched NK cells was assessed by flow cytometry using anti-CD56-PE, anti-CD3-FITC (Coulter Immunotech) antibodies, achieving 97% purity. NK cells were washed and plated in 24-well culture-plates.

### Immunoprecipitation

We further determined whether the recognition of LPG by TLR2 in NK cells also led to binding of TLR1 and/or TLR6 and additionally analyzed the activation pathway of TLR2 in NK cells of control subjects by immunoprecipitation of TLR2-MyD88, MyD88-IRAK-1, MyD88-TRAF-6, IRAK-1-TRAF-6 and TRAF-6-IKK-α. After purification, NK cells were suspended in RPMI-1640 with 10% heat-inactivated FBS and incubated for 2 h at 37°C with 5% CO_2_. Thereafter, 10×10^6^ NK cells were incubated with LPG (10 µg/mL) for 1 h at 37°C, 5% CO_2_ and the same number of NK cells were incubated in RPMI alone. Cells were washed twice with cold PBS and lysed in 250 µl modified radioimmunoprecipitation (RIPA) buffer (Tris-base, pH 7.4 10 mM, NaCl 150 mM, EDTA 1 mM, NaF 10 mM, NP-40 1%, PMSF 1 mM, aprotinin 10 mg/mL, leupeptin 1 mg/mL, Na_3_VO_4_ pH = 10 10 mM, DTT 1 mM) and incubated for 30 min on ice. Cell lysates were centrifuged at 10 000×*g* for 10 min at 4°C and the supernatants were collected. Protein concentration was determined using DC Protein Assay Reagents Package (Bio-Rad Laboratories, Hercules, CA, USA).

For immunoprecipitation assays, 200 µg of supernatants from LPG-activated and non-activated NK cells were pre-cleared with protein G-agarose beads (Life Technologies) for 1 h under agitation at 4°C and centrifuged at 14 000×*g* for 5 min at 4°C. Protein lysates were immunoprecipitated with anti-TLR2, anti-MyD88, anti-IRAK-1 or anti-TRAF-6 1∶20 (Santa Cruz antibodies: sc-10739, sc-11356, sc-7883, sc-8409, respectively) under agitation overnight at 4°C. The immunocomplex was captured with protein G-agarose beads for 2 h under agitation at 4°C. Beads were washed ten times in cold washing buffer (Tris-base, pH 7.4 10 mM, NaCl 150 mM, EDTA 1 mM, NP-40 1%) and immunoprecipitated proteins were diluted into 2× reducing Laemmli buffer pH = 6.6 (4 mL 10% SDS; 2.5 mL 0.5 M Tris-HCl, 0.4% (w/v) SDS; 1 mL 2-mercaptoethanol and glycerol 20%), boiled at 95°C for 5 min and stored at −70°C until Western blot assays were done.

### Total and nuclear protein extraction

To obtain the total protein extract, 2×10^6^ NK cells were incubated with LPG 10 µg/mL for 15, 30, 45 and 60 min or for 15 min with PMA. Cells were washed twice with PBS and lysed in 50 µl of RIPA modified buffer for 30 min. Cellular extracts were centrifuged at 10 000×*g* for 10 min at 4°C and the supernatants were collected. For nuclear proteins extracts 3×10^6^ NK cells were incubated with LPG 10 µg/mL for 1 h. Cells were washed twice with PBS and lysed by incubating them for 10 min in a detergent-free hypotonic buffer (10 mM Tris, pH = 7.6. 10 mM NaCl, 1.5 mM MgCl_2_, 0.5 mM EDTA, 1 mM DTT, 1.5 µg/mL leupeptin, 0.7 mM PMSF). Extracts were centrifuged at 4°C for 10 min at 956×*g*. The supernatants were discharged and intact nuclei were incubated in extraction buffer (20 mM Tris, pH = 8.0, 450 mM KCl, 0.05 mM EDTA, 1 mM DTT, 1.5 µg/mL leupeptin, 5 mM spermidine, 25% glycerol) for 45 min under constant agitation at 4°C. DNA pellets were eliminated by centrifugation for 15 min at 13 500×*g* at 4°C. Total and nuclear protein extracts were quantified with DC Protein Assay Reagents Package.

### Immunoblotting

For Western-blotting, 20 µg of total protein or nuclear extracts from non-stimulated and LPG-stimulated NK cells were used. Immunoprecipitates, nuclear and total protein extracts were analyzed by SDS–PAGE in 10% acrylamide gels. The proteins were transferred onto Immobilon-P membranes using a semidry electroblotting apparatus. The membranes were blocked with 5% milk in Tris-buffer saline-Tween 20 (TBST: 10 mM Tris–HCl, pH 7.4, 0.15 M NaCl, and 0.05% Tween 20) for 1 h at RT. Immunoblotting was done with anti-TLR1 (sc-8687), anti-TLR6 (sc-30001), anti-actin (sc-1616), anti-MyD88 (sc-11356), anti-IRAK1 (sc-7883), anti-TRAF6 (sc8409), anti-IKK-γ (sc-7181, 7330 and 8330), anti-pIκB-α (Cell signaling 9246), anti-pIKK-α/β (Cell signaling 2697) and anti-NF-κB p50 and p65 (sc-7178 and sc-372) diluted 1∶200 (sc- antibodies) or 1∶1000 (Cell signaling antibodies and actin) in TBST at 4°C overnight with constant shaking. HRP anti-rabbit IgG (E22, Biomeda), goat anti-mouse diluted 1∶5000 or bovine anti-goat IgG diluted 1∶10 000 in 5% non-fat dry milk were used as secondary antibodies, and incubated at RT for 1 h with shaking. Blots were developed using SuperSignal West Pico Chemiluminescent Substrate (Thermo Scientific) and exposed to X-ray films.

### Analysis of TLR expression by flow cytometry

NK cells (1×10^6^) from LCL and DCL patients as well as from healthy controls were incubated with 10 µg/mL LPG or with other TLR2 agonists such as PGN (10 µg/mL) or Pam_3_Cys-Ser (10 µg/mL) (EMC Microcollections GmbH, Tübingen, Germany) in 24 well culture-plates during 18 h in RPMI-1640 supplemented with 10% heat inactivated FBS at 37°C with 5% CO_2_. After washing with PBS, LPG-stimulated NK cells, as well as non-stimulated cells, were fixed with 2% paraformaldehyde (Merck) and incubated with blocking buffer (PBS containing human IgG, 2% FBS, 5 mM EDTA and 0.1% sodium azide) on ice for 30 min. Afterwards the cells were washed and stained with 2 µL of Ab goat anti-human TLR2, TLR1 or TLR6 (sc- 8680, sc-8687, sc-30001) and FITC conjugated rabbit anti-goat IgG (Zymed 81-1611) or isotype controls. Additionally, cells were incubated with anti-CD56-PE (BD-Pharmingen).

TLRs were analyzed in 10^4^ NK cells by FACS, using CellQuest software (FACScan BD Immunocytometry Systems, Mountain View, CA. USA). Results are expressed in MFI. For some experiments, NK cells were stained with anti-CD56, anti-CD16, anti-TLR2, anti-TLR1 or anti-TLR6 for 30 min at RT. The antibodies used for this analysis included: PE-conjugated anti-CD56, PE-Cy^TM^5-anti-CD16, PE-Cy^TM^5 mouse (IgG1k) and PE mouse (IgG1k) for isotype controls, all from BD-Pharmingen; rabbit polyclonal IgG TLR6 (H-90), goat polyclonal IgG TLR1 (N-20), goat polyclonal IgG TLR2 (N-17) all from Santa Cruz Biotechnology; FITC rabbit anti-goat IgG (H+L) conjugated, FITC goat anti-rabbit IgG (H+L) from Zymed and FITC goat IgG isotype control from Coulter.

### Immunohistochemistry (IHC) for detection of NK cells, TLRs and cytokines in NK cells from LCL and DCL patients

Skin punch biopsies (4–6 mm) were taken from the lesions of DCL and LCL patients. The tissues were embedded in paraffin, cut into 5-µm thick slices. Some of the slides were stained with H&E to evaluate the inflammatory characteristics. For Immunohistochemistry analysis the slides were hydrated and antigenically reactivated in a citrate buffer (0.01 M citric acid, 0.01 M sodium citrate) for 10 min at 95°C. Endogenous peroxidase was blocked with methanol/H_2_O_2_ 3% for 10 min and nonspecific antigenic sites were blocked with 3% bovine serum albumin dissolved in Tris-HCl pH = 7.6 with 0.1% Triton X-100 for 60 min at RT. Thereafter, samples were stained with mouse anti-CD57 (Zymed 08–0167) overnight at 4°C, washed, incubated with secondary antibody biotin anti-mouse (Zymed 62-6540) for 30 min at RT and with streptavidin AP (AB complex/AP, DAKO K0376,) for 30 min at RT. Tissues were washed and color development was assessed after incubation with AP Red substrate kit (Zymed 00–2203) or Stay Green/AP kit (Abcam antibodies: ab-156428) at RT.

TLRs and cytokines were detected by double staining. For this, the samples were washed and endogenous peroxidase and nonspecific antigens were blocked as described above. Thereafter, the samples were incubated 1∶50 with goat anti-TLR1, mouse anti-TLR2, rabbit anti-TLR6, mouse anti-IFN-**γ** or mouse anti-TNF-α 1∶100 (sc-8687, sc-21759, sc-30001, ab-11866 and sc-1350, respectively) for 30 min at RT, washed and secondary antibodies were incubated for 30 min at RT. The secondary antibodies included: mouse and rabbit specific HRP/AEC detection IHC kit (Abcam ab94705) for TLR6, biotin-labelled rabbit anti-goat (Zymed 81–1640) antibodies for TLR1 and TNF-α and biotin-labelled goat anti-mouse (Zymed 62–6540) was used for detecting TLR2 and IFN-**γ**. Thereafter, tissues were washed and incubated with horseradish peroxidase (Zymed 43–4311) for 30 min at RT. For TLR and IFN-γ detection samples were washed and color development was assessed after incubation with DAB Black kit (Biocare Medical BRI40 H, L). For TNF-α, a DAB Substrate kit was used (Roche Cat. 11718096001). The slides were counterstained with Mayeŕs haematoxylin (Biogenex, CA, USA). Digital images of tissue sections were captured using a light microscope and an AxioCam MRc5 camera (Zeiss, Germany). In order to obtain the number of single and double positive cells in these lesions, cells were counted in 8 pictures of each tissue were taken with a final area corresponding to 1 mm^2^ of 3 LCL and 3 DCL patients. Controls for primary and secondary antibodies were negative.

### IFN-γ and TNF-α production

NK cells (1×10^6^) from LCL and DCL patients, as well as from healthy donors were incubated with 10 µg/mL LPG in 1 mL RPMI-1640 with 10% heat-inactivated FBS during 18 h at 37°C and 5% CO_2_. IFN-γ and TNF-α were analyzed by ELISA tests in 96-well plates (Costar, Corning, NY). Samples were set up in triplicates. Briefly, microtiter plates were coated with anti-TNF-α (clone MAb1, 6 µg/mL; BD Pharmingen, San Diego, CA) or anti-IFN-γ (clone NIB42, 6 µg/mL; BD Pharmingen) in 100 mM Na_2_HPO_4_, pH 9.0 during 12 h at 4°C and blocked with PBS containing 0.05% Tween 20 and 10% FBS. The supernatants and recombinant hTNF-α (BD Pharmingen) or recombinant hIFN-γ (R&D Systems) were incubated with RPMI-1640 medium containing 10% FBS during 2 h at RT. Both cytokines were detected with biotinilated anti-hTNF-α (clone MAb11, 2 µg/mL; BD Pharmingen) or anti-hIFN-γ (clone 4S.B3, 2 µg/mL; BD Pharmingen) in 1% BSA for 1 h. The plate was developed using streptavidine-alkaline-phosphatase conjugate (Life Technologies) with p-nitrophenyl phosphate (4 mg/mL, Life Technologies) as substrate. Absorbance was measured at 405 nm with an ELISA reader (BIO-TEK INSTRUMENTS). The detection limits for both cytokines were 15 pg/mL.

### Gene expression of TLR2, IFN-γ and TNF-α by Real-time PCR

Gene expression of TLR2, IFN-γ and TNF-α were analyzed by real-time PCR in 5 healthy controls, 6 LCL and 3 DCL patients. Total RNA from non-stimulated and LPG-stimulated NK cells (18 h) was retro-transcribed using High-Capacity cDNA Archive kit (Applied Biosystems), according to manufactureŕs instructions. Quantitative Taqman PCR analysis was performed with the ABI PRISM 7900HT Sequence Detection System (Applied Biosystems) containing 1× Taqman Universal Master Mix (Applied Biosystems) and 1× probes and primers sets Hs00610101_m1 (TLR2), Hs00174128_m1 (TNF) and Hs00989291_m1 (IFN). The thermal profile was as follows: 95°C for 10 min and 40 cycles at 95°C for 15 s and 60°C for 1 min. All amplification reactions were done in duplicate and the relative quantification of TLR2, TNF-α and IFN-γ gene expression were calculated using the comparative Ct method (2^−ΔΔ*C*T^) [16]. Levels of mRNA expression were assessed after normalization, using GAPDH as internal control.

### Statistical analysis

Statistical differences between groups were obtained using Mann-Whitney U-test or Student's T-test. The Kruskal-Wallis test was used for the comparison of more than two groups of data. Correlation analyses were performed by Spearmańs test. Data are presented as mean ±SEM, *p*<0.05 was considered statistically significant. These statistical analyses were done using the Prism 5 software (GraphPad Software, San Diego, CA, USA).

## Results

### LPG bindsTLR1 and TLR6 and activates the TLR signaling pathway

**Figure 1:**
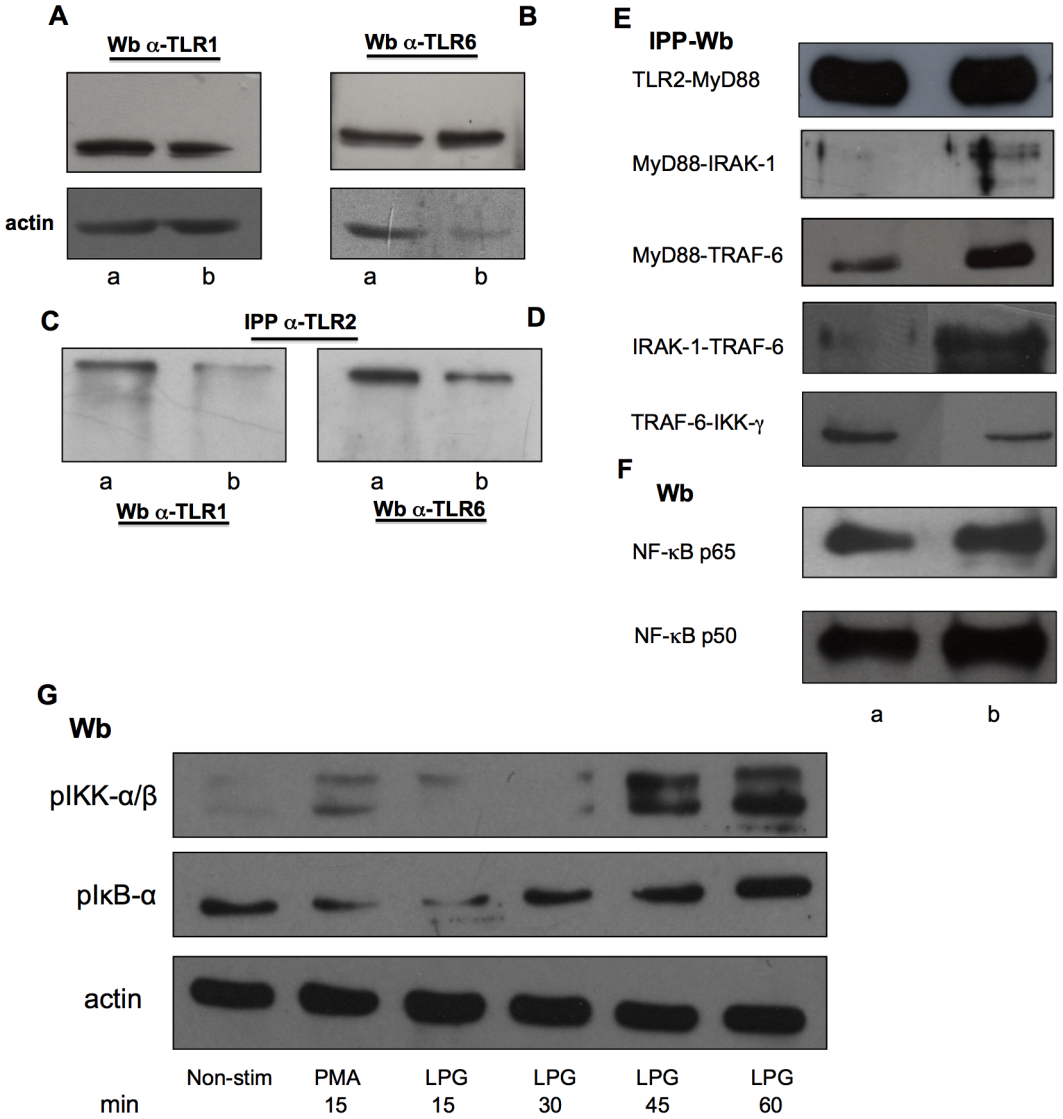
LPG promotes binding of TLR signaling proteins and NF-κB nuclear translocation. (A and B) Western blot shows TLR1 and TLR6 in NK cells. (C and D) Immunoprecipitations were performed with anti-TLR2 in non-stimulated (lane a) and LPG-stimulated NK cells after 1 h of co-incubation (lane b). (C) Immunoprecipitates were subjected to Western blotting and probed with anti-TLR1 and (D) anti-TLR6 antibodies, respectively. A representative immunoblot from four different experiments is shown. (E) NK cell lysates in non-stimulated and LPG-stimulated NK cells were immunoprecipitated with anti-TLR2, anti-MyD88, anti-IRAK-1 and anti-TRAF-6 antibodies and Western blotted with anti-MyD88, anti-IRAK-1, anti-TRAF-6 and anti-IKK-γ antibodies. (F) Nuclear translocation of p50 and p65 NF-κB isoforms were analyzed in non-stimulated and LPG-stimulated NK cells. (G) Phosphorilation of pIKK-α/β and pIκB-α were analyzed in non-stimulated NK cells (Non-stim) or in cells stimulated with PMA (15 min) or LPG (15, 30, 45 and 60 min). A representative immunoblot from two different experiments is show.

Both TLR1 and TLR6 proteins are expressed in non-stimulated and LPG-stimulated NK cells from healthy controls (Fig. 1A and 1B). In order to analyze whether TLR2 binds to TLR1 and TLR6 in NK cells, we immunoprecipitated with anti-TLR2 and Western blotted with α-TLR1 or α-TLR6. A recognition band was found both experimental conditions: in non-stimulated and LPG-stimulated NK cells. It is noteworthy, that in both cases, a more intense recognition was observed in non-stimulated NK cells (Fig. 1C and 1D, lane a) as compared to LPG-stimulated NK cells (Fig. 1C and 1D, lane b). We speculate that the reduced binding of TLR1 or TLR6 to TLR2 after incubation with LPG is possibly due to partial obstruction of the docking site after LPG binds to TLR2.

Through immunoprecipitations we analyzed the binding of proteins involved in the TLR signaling pathway in non-stimulated and LPG-stimulated NK cells. The immunoprecipitations included: TLR2-MyD88, MyD88-IRAK-1, MyD88-TRAF-6, IRAK-1-TRAF-6 and TRAF-6-IKK-γ. We observed protein binding in all immunoprecipitations, with an increase when the NK cells were stimulated with LPG (Fig. 1E, second, third and fourth blots). Only the binding of TRAF-6-IKK-γ decreased (Fig. 1E, fifth blot). This decrease in binding was expected, since the kinases need to be degraded to induce NF-κB translocation to the nucleus. To determine whether LPG induces NF-κB nuclear translocation, we used nuclear extracts of NK cells to analyze p50 and p65 isoforms and their nuclear translocation. We found nuclear translocation of both isoforms in LPG-stimulated NK cells (Fig. 1F, lane b).

Finally, we analyzed the kinetics of phosphorylation of the kinases IKK and IκB in non-stimulated NK cells and in cells stimulated with PMA 10 µg/mL or LPG 10 µg/mL during 15, 30, 45 and 60 min. We observed an increase in phosphorylation of these kinases at 45 and 60 min after stimulation with LPG (Fig. 1G). With these data we were able to confirm that LPG activates NK cells through the TLR2 pathway.

### NK cells in peripheral blood and lesions of LCL and DCL patients

**Figure 2:**
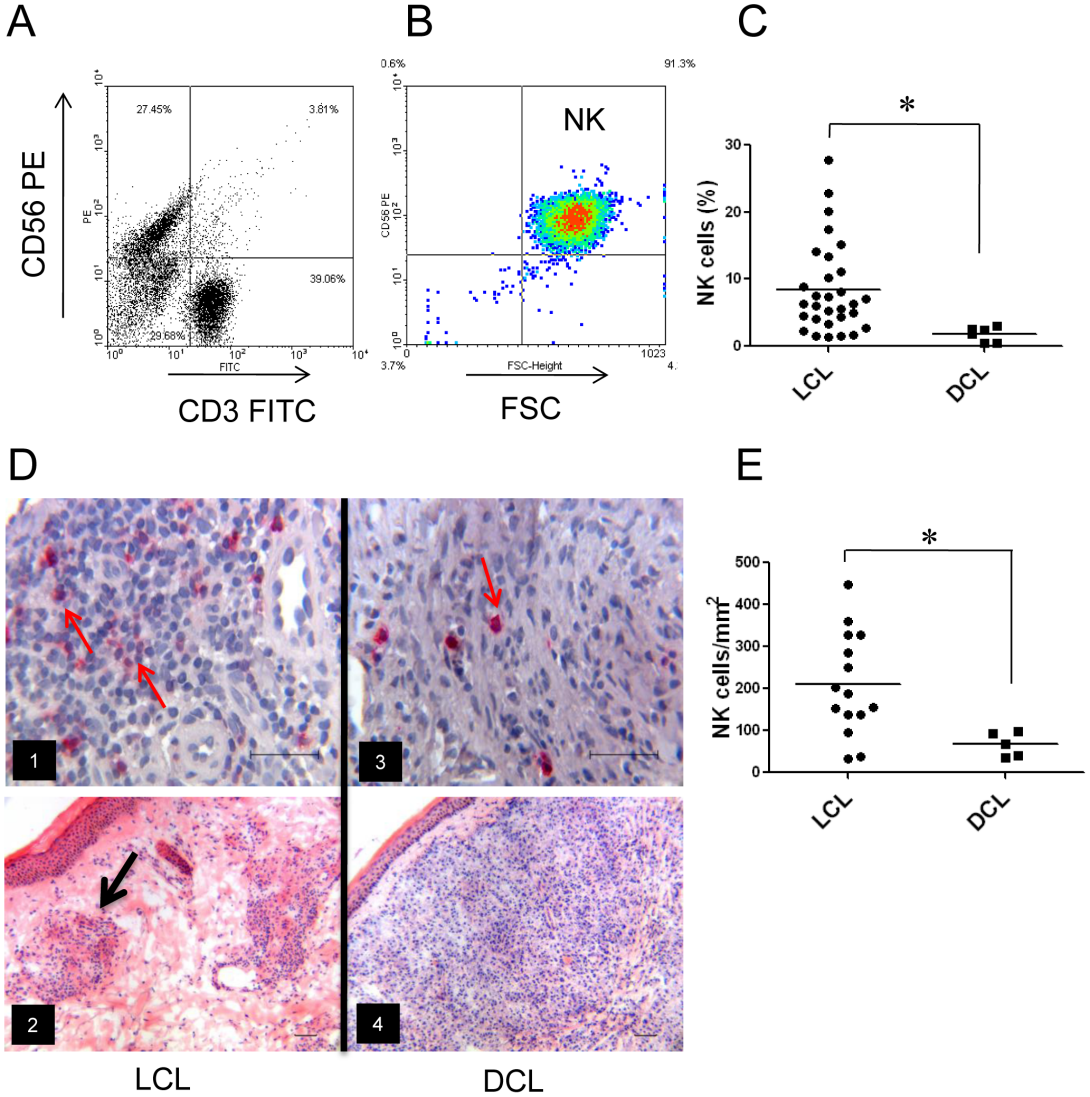
NK cells in patients infected with *Leishmania mexicana*. (A) Representative flow cytometry plots of peripheral blood from patients before separation of NK cells from PBMC. (B) NK cells after purification (CD56^+^/CD3^-^). (C) Percentage of NK cells from patients with cutaneous leishmaniasis in peripheral blood [LCL (n = 30) and DCL (n = 6)]. Each dot represents a patient and horizontal line represents the mean of each group. (D) Immunostaining of NK cells in lesions shown NK cells. (D1) NK cells in LCL patients stained in red (CD57^+^) some of which are marked with red arrows. (D3) NK cells in lesions of DCL patients. (D2) H&E staining of lesions from LCL patients showing granuloma (black arrow). (D4) H&E staining of lesion from DCL patient. (E) Number of NK cells per mm^2^ in lesions. Each dot represents a patient and the horizontal line represents the mean. Images are representative of LCL patients (n = 15) and DCL patients (n = 5). *p≤0.05 was considered significant. Scale bar  = 50 µm.

Quantitation of NK cells in blood and tissues of patients with both clinical forms revealed that LCL patients had more NK cells in blood as well as in lesions, as compared to DCL patients. NK cells in PBMC were analyzed by flow cytometry (Fig. 2A and 2B), showing that the percentage of NK cells in peripheral blood of LCL patients ranged from 1.4–27.8% (mean: 8.43±1.22), whereas in DCL patients they ranged from 0.5–3.1% (Fig. 2C). No correlation was found between the number of NK cells in peripheral blood and disease duration, age or gender.

Immunohistochemical stains of NK cells in skin biopsies showed enhanced numbers of NK cells in lesions of LCL patients, as compared to DCL patients (Fig. 2D images 1 and 3). H&E stains showed granuloma formations in LCL tissues (Fig. 2D, image 2), whereas in DCL patients, a randomly scattered distribution of inflammatory cells was found (Fig. 2D, image 4). NK-cell counts in tissues showed a mean number 210 cells/mm^2^ for LCL patients, whereas DCL patients showed 67 cell/mm^2^ (Fig. 2E). Taken together, LCL patients had significantly more NK cells as compared to DCL patients, both in peripheral blood as well as in infected lesions.

### TLR2 expression on NK cells of peripheral blood

**Figure 3:**
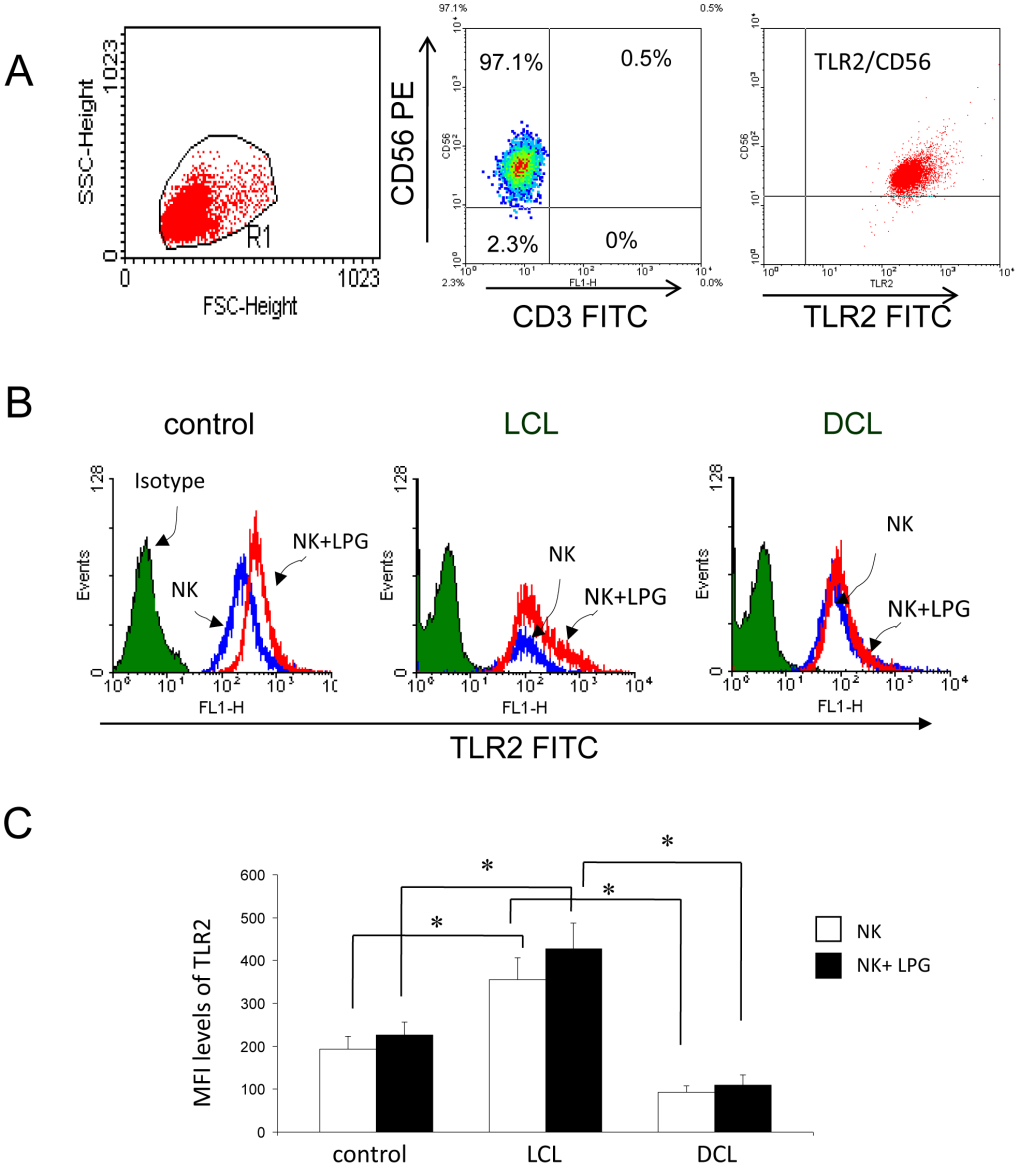
Analysis of TLR2 expression on NK cells. (A) Representative flow CD56^+^/CD3^-^ expression in membranes of NK cells; right image: NK cells were gated and analyzed for TLR2 (TLR2^+^/CD56^+^) expression. (B) Representative histograms showing the MFI levels of TLR2 expression on NK cells from control and patients (LCL and DCL). (C) Quantitation of TLR2 expression on NK cells. □ Non-stimulated cells, ▪ LPG-stimulated cells. Data are representative of healthy controls (n = 21), LCL patients (n = 28) and DCL patients (n = 6). These results are the mean ±SEM. *p≤0.05 was considered significant.

NK cells were isolated by negative selection achieving a purity of 97%, as shown by flow cytometry (Fig. 3A, middle image). TLR2 expression was analyzed in non-stimulated as well as LPG-simulated NK cells of 28 LCL patients, six DCL patients and 21 healthy controls. All NK cells expressed TLR2, albeit with different intensity. NK cells of LCL patients expressed significantly higher levels of TLR2, as compared to DCL cells or to healthy controls (Fig. 3B and 3C), yet no differences were found in TLR2 expression when comparing non-stimulated with LPG-stimulated NK cells within each patient group (Fig. 3C).

**Figure 4:**
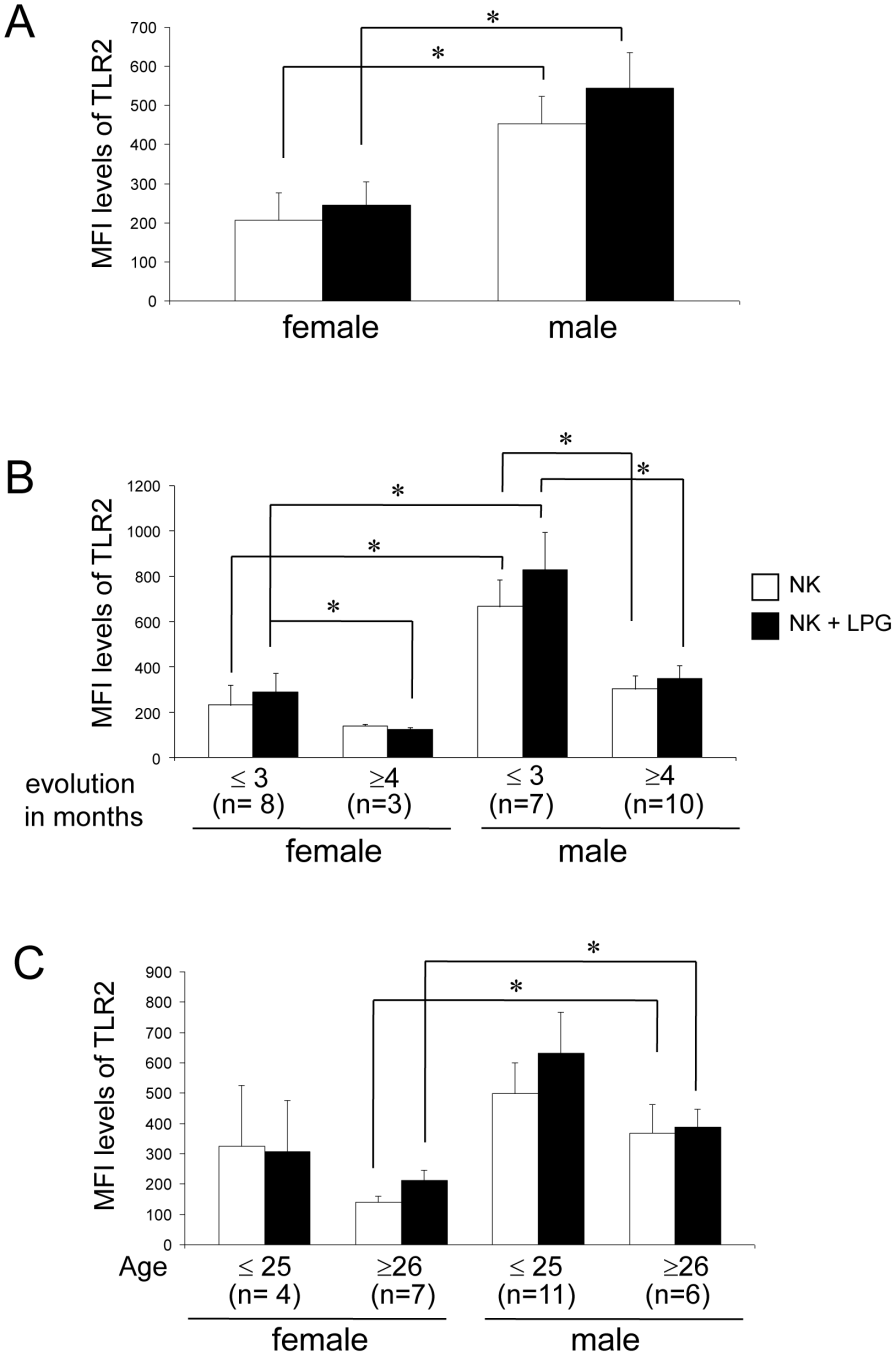
TLR2 expression in NK cells in LCL patients (n = 28). Analysis according to: (A) gender, (B) disease evolution (≤3 or ≥4 months) or (C) age (≤25 or ≥26 years). □ Non-stimulated NK cells, ▪ LPG-stimulated NK cells. Cell surface expression is indicated by MFI. These results are the mean ± SEM. *p≤0.05 was considered significant.

To ascertain whether the increased TLR2 expression found in LCL patients was related to gender, disease duration or age, we subdivided the 28 patients according to these parameters. Thus, we analyzed the TLR2 expression in 11 females and 17 males. We found that NK cells of male LCL patients expressed significantly higher levels of TLR2, as compared to females, both in non-stimulated as in LPG-stimulated cells (Fig. 4A). When analyzing TLR2 expression in NK cells according to disease progression, we found that both in males and females the expression of TLR2 diminishes significantly after four months of disease duration (Fig. 4B). The analysis of TLR2 expression according to age revealed that males ≥26 years always express higher levels of TLR2, as compared to female LCL patients of the same age group, both in non-stimulated as well as in LPG-stimulated cells (Fig. 4C).

### TLR1, TLR2 and TLR6 expression on purified NK cells of LCL and DCL patients

Having shown that LPG is a ligand for TLR1, TLR2 and TLR6 and that TLR2 can bind with either TLR1 or TLR6, we analyzed whether specific heterodimers could be associated with the clinical forms of the disease. We analyzed the expression of all three TLRs in non-stimulated and LPG-stimulated NK cells in three groups of individuals: nine LCL patients (two females and seven males, who had a mean age 25±5 years, and a mean disease duration of five months); four DCL patients (one female and three males, who had a mean age of 52 years, and a disease duration 19 years), and four healthy controls without a history of leishmaniasis.

**Figure 5:**
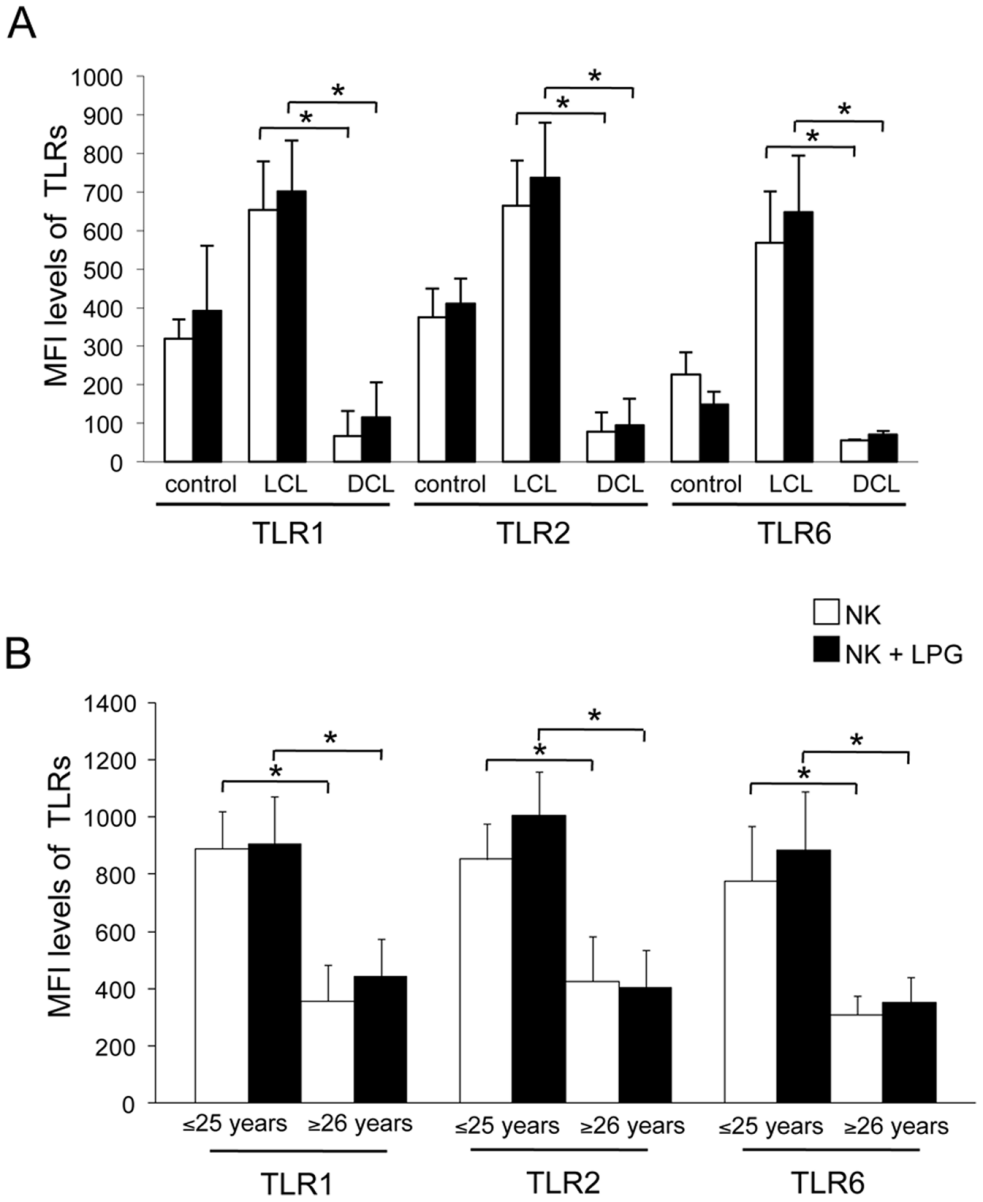
TLR1, TLR2 and TLR6 expression on NK cells from controls and patients (LCL and DCL). (A) Analysis according to disease form. (B) TLR2, TLR1 and TLR6 expression according to age (≤25 or ≥26 years) in LCL patients. □ Non-stimulated NK cells, ▪ LPG-stimulated NK cells. Cell surface expression is indicated by MFI. These results are the mean ±SEM. *Significant differences were observed between LCL and DCL patients for all TLRs in LPG-stimulated and non-stimulated cells.

Healthy controls and LCL patients expressed significantly higher levels of all three TLRs, as compared to DCL patients. LPG stimulation showed no significant increase in TLR expression between LCL and DCL patients (Fig. 5A).

Only LCL patients were analyzed for TLR1, TLR2 and TLR6 expression, according to age, due to the reduced number of DCL patients. The nine LCL patients were divided into in two groups: ≤25 years (n = 5) and ≥26 years (n = 4). A significant reduction of all three TLR receptors was observed in patients ≥26 years, as compared to patients ≤25 years, since the later expressed only half the values of TLR1, TLR2 and TLR6, both in non-stimulated as well as in LPG-stimulated NK cells (Fig. 5B).

In an attempt to analyze whether this non-responsiveness of all three receptors was specific for LPG, we analyzed the expression of these TLRs in NK cells of both groups of patients after stimulation with additional TLR2 agonists: PGN (peptidoglycan) and Pam_3_Cys-Ser. Significant differences in the expression of all three receptors on NK cells were observed between the two patient groups: whereas NK cells of LCL patients showed an enhanced expression of all three TLR receptors, all of which tended to increase with TLR2 agonists, DCL patients showed significantly lower levels of TLR1, TLR2 and TLR6 expression, which remained unchanged despite stimulation with various TLR2 ligands (S1). These data show that the unresponsiveness of NK cells of DCL patients is not specific for *Leishmania* LPG, but for the other TLR2 agonists as well.

### TLR1, TLR2 and TLR6 expression on NK cells of tissue lesions of LCL and DCL patients

**Figure 6:**
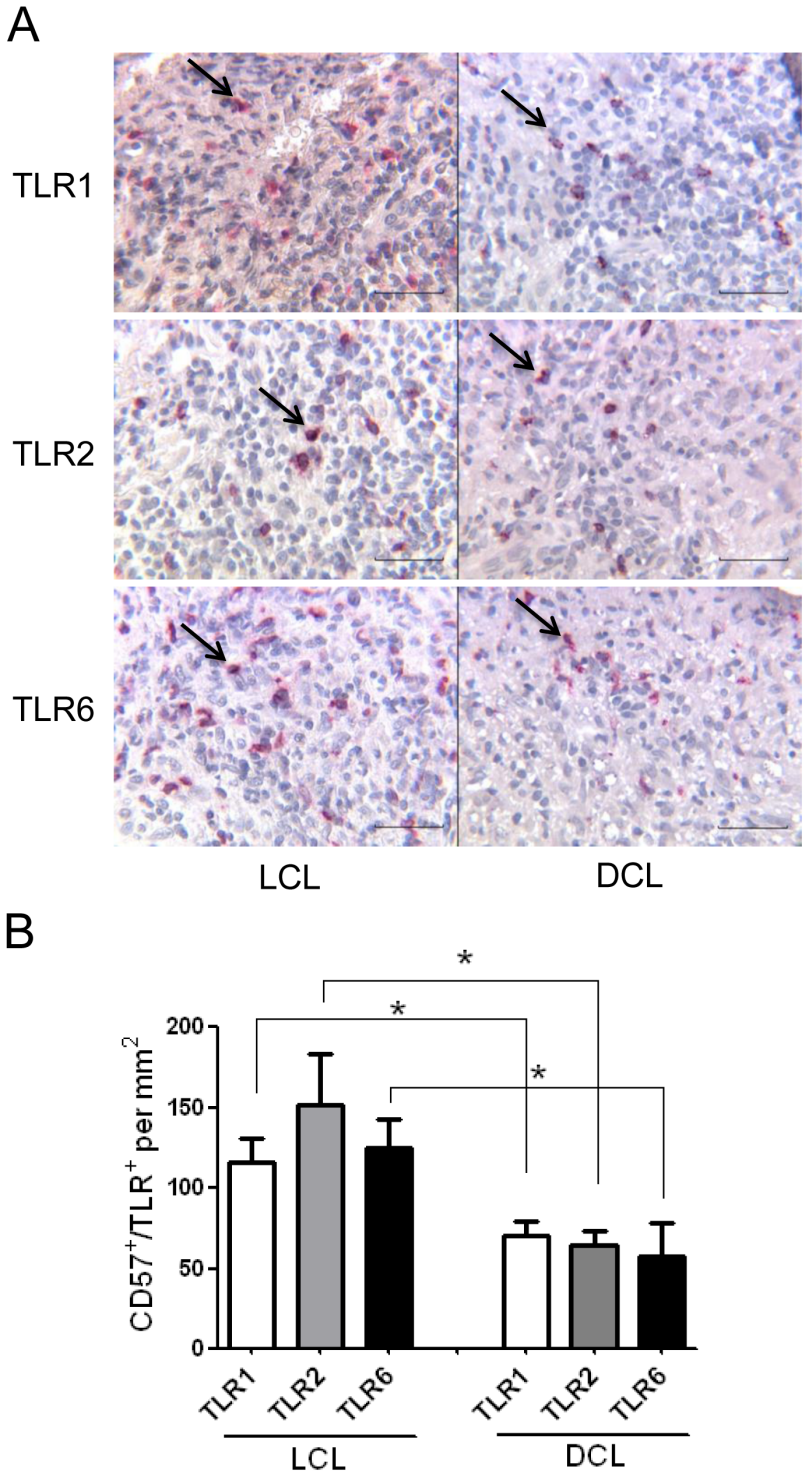
TLR1, TLR2 and TLR6 expression on NK cells in lesions of 3 LCL and 3 DCL patients. (A) Double immunohistochemistry staining of TLR expression (TLR1, TLR2 and TLR6) on NK cells (CD57). (B) Number of NK cells expressing TLRs per mm^2^. Results are the mean ±SEM. Scale bar  = 50 µm. Black arrows show double positive cells.

To assess the phenotype and distribution of NK cells expressing these TLRs in the lesions of LCL and DCL patients, we performed double immunostaining: CD57 and TLR1 (or TLR2 or TLR6) (Fig. 6A). The mean number of NK cells expressing TLR1, TLR2 or TLR6 in LCL patients was 115±l5, 151±31 and 125±17 cells/mm^2^, whereas in DCL patients the mean number was 70±8, 65±8 and 58±20 cells/mm^2^, respectively (Fig. 6B). Thus, LCL patients showed significantly higher numbers of NK cells (CD57^+^/TLR^+^) expressing TLR2, TLR1 or TLR6 on NK cells, as compared to DCL patients.

### TLR1, TLR2 and TLR6 expression in NK subsets CD56^dim^ and CD56^bright^

**Figure 7:**
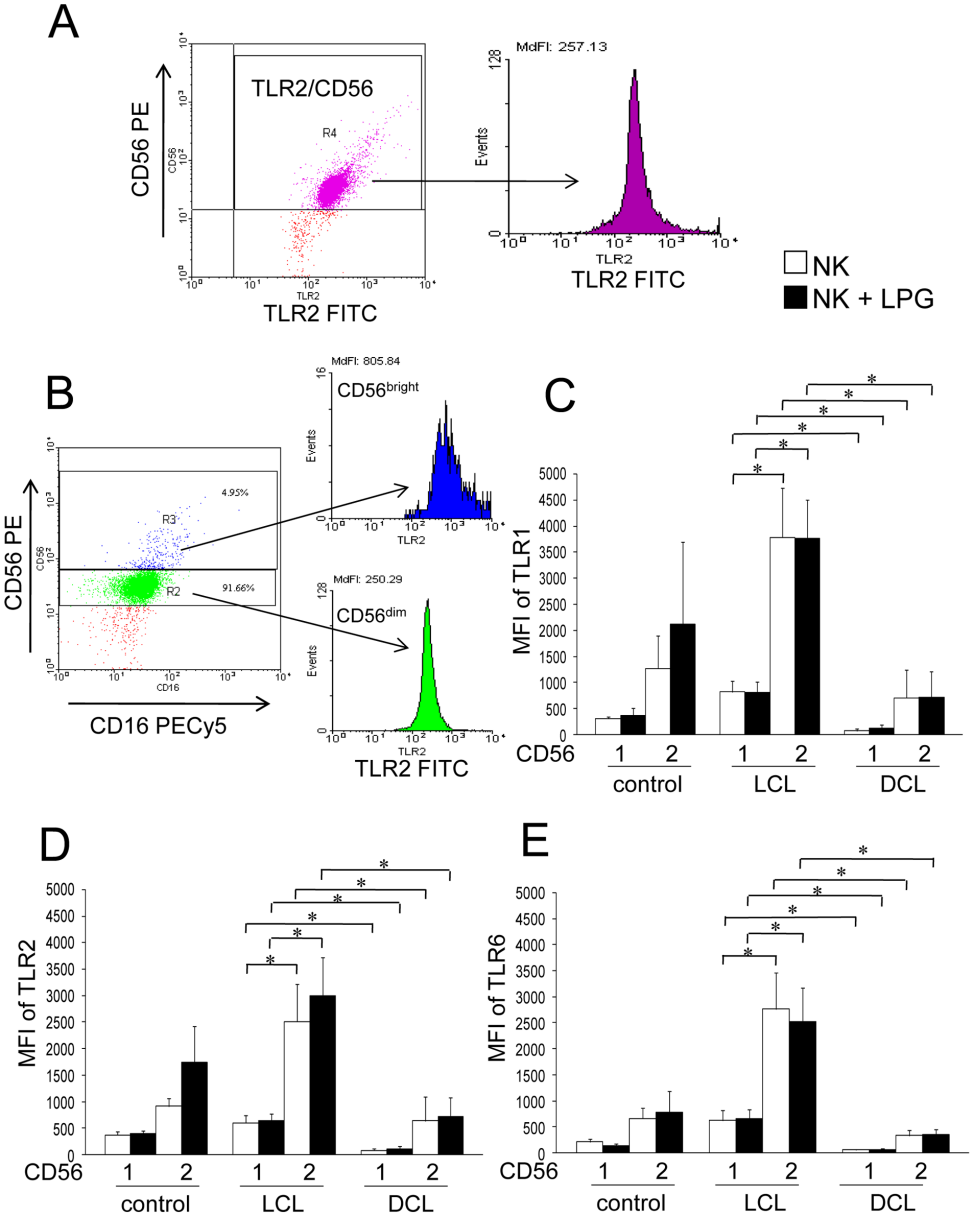
TLR1, TLR2 and TLR6 expression on NK-cell subsets (CD56^bright^ and CD56^dim^) of patients and controls. (A) Representative flow cytometry dot plot (TLR2 vs. CD56) and histogram of TLR2 expression in NK cells. (B) This gate was subsequently analyzed for TLR2 expression on NK-cell subsets. Dot plot (CD16^+^/CD56^+^) and histogram for CD56^bright^ (blue box) and CD56^dim^ (green box) NK cells. (C) TLR1 expression. (D) TLR2 expression. (E) TLR6 expression. □ Non-stimulated NK cells; ▪ LPG-stimulated NK cells. Lane 1: CD56^dim^; lane 2: CD56^bright^ NK cells. Cell surface expression is indicated by MFI. Results are the mean ±SEM. *Significant differences were observed between NK cell subsets of LCL and DCL patients for all 3 TLRs in LPG-stimulated and non-stimulated cells. LCL patients (n = 9), DCL patients (n = 4) and controls (n = 4).

Since NK cells are subdivided phenotypically according to their function into CD56^dim^ (cytotoxic) and CD56^bright^ (cytokine producing) cells, we were interested in analyzing TLR1, TLR2 and TLR6 expression in both NK subsets of LCL and DCL patients (Fig. 7A and 7B). We therefore analyzed the expression of these receptors in 9 LCL and 4 DCL patients, before and after stimulation with LPG. Only NK CD56^bright^ cells expressed high levels of TLR1, TLR2 and TLR6, which were significantly higher in LCL patients (4 to 8-fold), as compared to DCL patients. There were also significant differences in TLRs expression between both NK subsets: CD56^dim^ expressed significantly lower levels of TLRs as compared to CD56^bright^ (Fig. 7C, 7D and 7E). However, no significant differences were found between non-stimulated and LPG-stimulated NK cells in both subsets of NK cells of the different groups.

### IFN-γ and TNF-α production by NK cells in blood and tissue lesions

**Figure 8:**
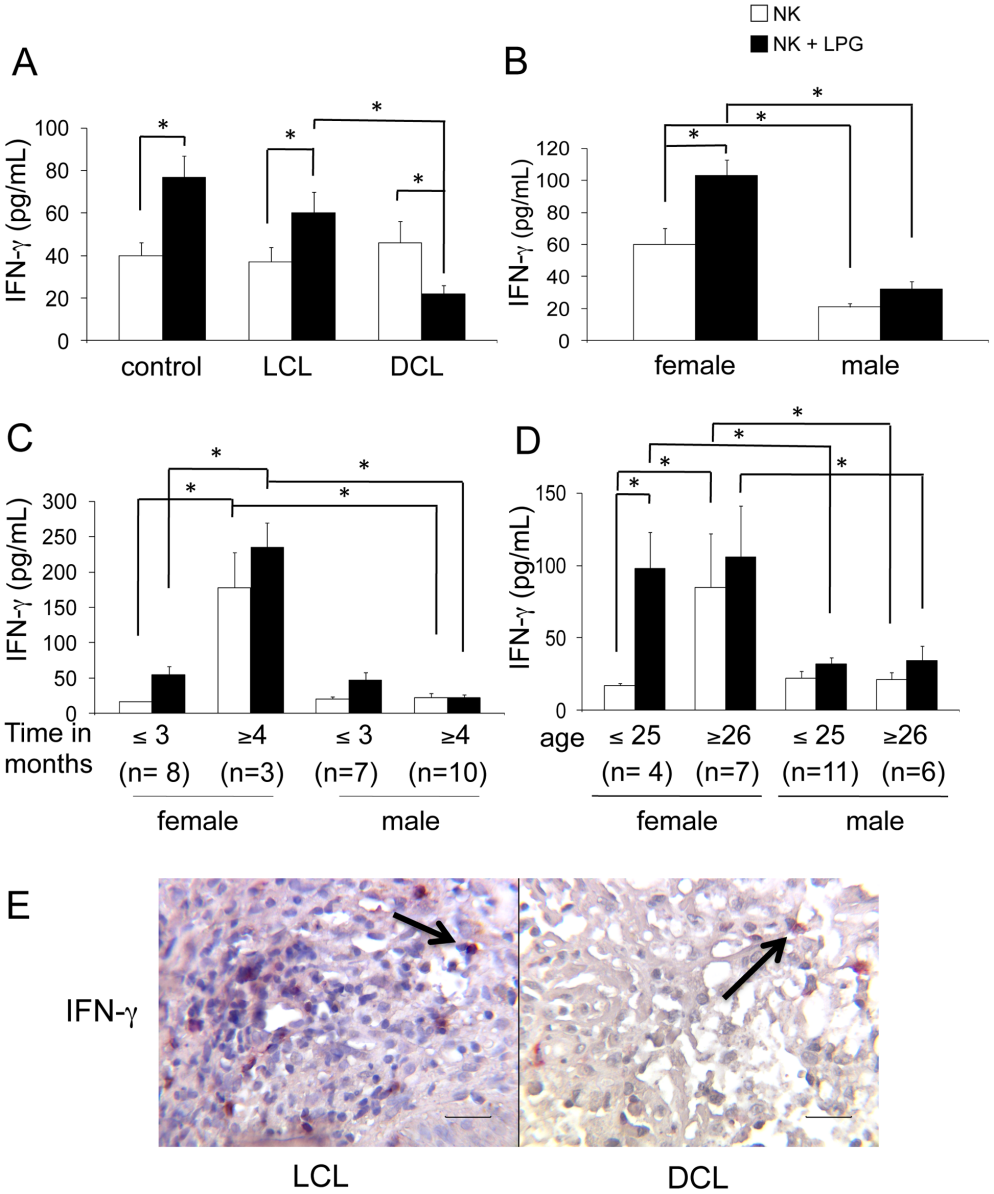
IFN-γ production by NK cells. (A) IFN-γ production of peripheral blood NK cells [control subjects (n = 21), LCL patients (n =  28) and DCL patients (n = 6)]. Analysis in LCL patients [female (n = 11) and male (n = 17)] according to: (B) gender; (C) disease duration; (D) age. (E) Double immunohistochemical labelling (CD57^+^/IFN-γ^+^) in lesions of patients (LCL and DCL) showed redish-brown staining generated by the combination of a red AP substrate used for NK cells and DAB Black used for IFN-γ staining. □ Non-stimulated NK cells. ▪ LPG-stimulated NK cells. Mean ±SEM is shown. *p≤0.05 was considered significant. Scale bar  = 50 µm. Black arrows show double positive cells.

Production of IFN-γ and TNF-α was analyzed in 28 LCL, 6 DCL patients and 21 healthy donors in LPG-stimulated and non-stimulated purified NK cells. Non-stimulated NK cells from healthy subjects and LCL, DCL patients produced similar basal amounts of IFN-γ (with mean values of: 39, 37, 46 pg/mL, respectively). Yet after stimulation with LPG, only NK cells from LCL patients and healthy subjects increased their IFN-γ production (mean values: 60 and 77 pg/mL, respectively). In contrast, NK cells from DCL patients reduced their IFN-γ production to half their basal value (from 46 to 22 pg/mL), when stimulated with LPG (Fig. 8A). To ascertain whether the increased IFN-γ production found in LCL patients was related to gender, disease duration and/or age, we subdivided the 28 patients according to these parameters. Thus, we analyzed the cytokine production in 11 females and 17 males. Female patients produced significantly more IFN-γ in non-stimulated (60 pg/mL) and LPG-stimulated (103 pg/mL) NK cells, as compared to males, which only showed a slight increase in IFN-γ production (21 to 32 pg/mL) after LPG stimulation (Fig. 8B). In an attempt to associate IFN-γ production with disease evolution in female and male LCL patients, we further separated the patients into groups according to their disease duration into ≤3 months or ≥4 months. NK cells of female LCL patients with ≥4 months disease duration showed higher IFN-γ production in basal conditions, as well as after LPG stimulation, as compared to those with ≤3 months or to male LCL patients (Fig. 8C). When analyzing IFN-γ production according to age, we found that females, particularly those aged ≤25 (37% of the women) showed a vigorous response (5-fold increase) when NK cells are stimulated with LPG. Although NK cells of female LCL patients aged ≥26 showed a higher IFN-γ production in non-stimulated NK cells, as compared to younger females, these cells responded only slightly to LPG. This stands in contrast to the minimal response towards this parasite antigen found in male patients of any age, who only showed a minimal increase in cytokine production after LPG stimulation (Fig. 8D). Taken together, our data reveal that NK cells of female patients aged ≤25 years, with disease duration of ≥4 months, showed the most vigorous IFN-γ production when the cells are stimulated with LPG, whereas NK cells from female patients aged ≥26 years already come activated and therefore respond only weakly to further stimulus by LPG. This contrasts with the diminished IFN-γ production in male NK cells of all age groups, in both non-stimulated and LPG-stimulated conditions, irrespective of disease duration. When comparing IFN-γ production in NK cells of lesions in LCL and DCL patients, we found that LCL patients showed higher numbers of double positive cells (CD57^+^/IFN-γ^+^), as compared to DCL patients (Fig. 8E). Our data show that IFN-γ production by NK cells in both blood and tissue lesions were markedly reduced in DCL, as compared to LCL patients.

**Figure 9:**
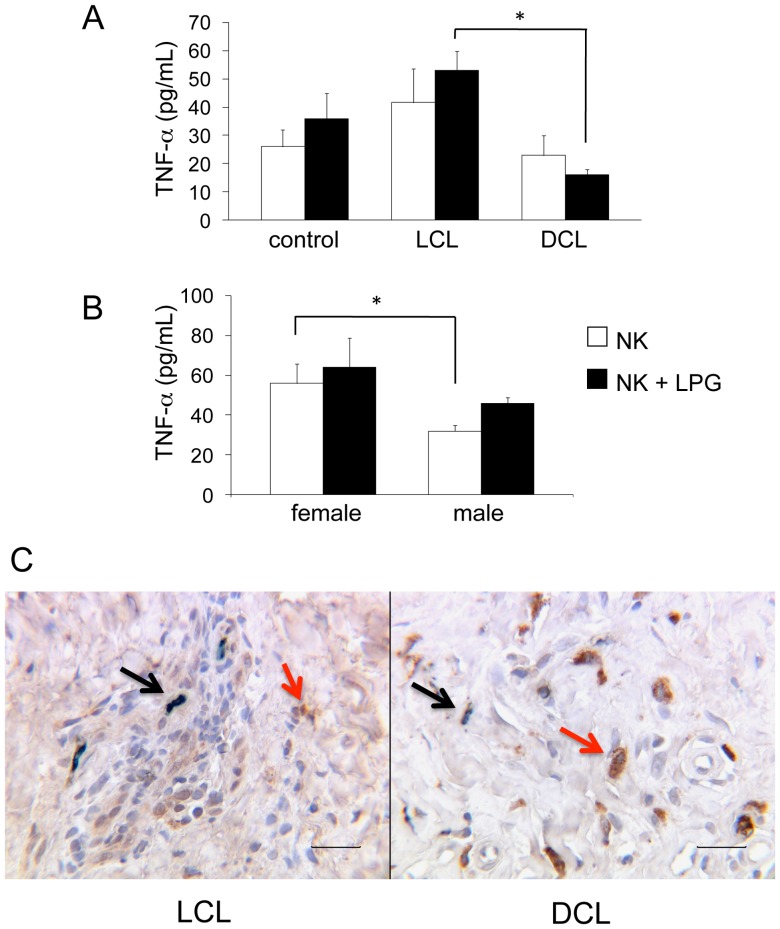
TNF-α production by NK cells. (A) TNF-α production in peripheral blood [control subjects (n = 21), LCL patients (n =  28) and DCL patients (n = 6)]. (B) Analysis of TNF-α production of LCL patients according to gender [female (n = 11) and male (n = 17)]. (C) Double immunohistochemistry (CD57^+^/TNF-α^+^) staining of lesions of LCL and DCL patients showed dark green staining induced by the combination of a green substrate (Stay Green/AP) used for NK cells and DAB (brown) used for TNF-α staining. □ Non-stimulated NK cells, ▪ LPG-stimulated NK cells. Mean ±SEM is shown. *p≤0.05 was considered significant. Scale bar  = 50 µm. Double positive cells CD57^+^/TNF-α^+^ (black arrows) and single positive cells CD57^-^/TNF-α^+^ (red arrows).

Additionally, we analyzed TNF-α production in NK cells in the same group of subjects and found that LCL patients produced higher levels (mean: 42 pg/mL) as compared to DCL patients (mean: 23 pg/mL), particularly after LPG stimulation, where, instead of enhancing cytokine production as in LCL patients (53 pg/mL), NK cells of DCL patients further reduced their TNF-α production (17 pg/mL). The difference of TNF-α production in LPG-stimulated NK cells between LCL and DCL patients was significant (Fig.9A). We also analyzed TNF-α production in LCL patients according to gender, disease duration and age. NK cells of female LCL patients produced significantly more TNF-α than male LCL patients in non-stimulated (56 vs 32 pg/mL) cells. After LPG stimulation, NK cells of both females and males increased their TNF-α production (64 vs 46 pg/mL), albeit the differences were not statistically significant (Fig. 9B). No significant difference in TNF-α production was found when comparing different age groups or disease duration (data not shown). The analysis of TNF-α production in lesions of both patient groups showed that LCL patients had more double positive cells (CD57^+^/TNF-α^+^), as compared to DCL patients (Fig. 9C, black arrows). Interestingly, many large single positive cells (CD57^-^/TNF-α^+^) are observed in tissues of DCL patients (Fig. 9C, red arrows). Since they are not NK cells, the exact nature of these larger cells withTNF-α^+^ staining found in tissues of DCL remains to be determined.

### TLR2, IFN-γ and TNF-α gene expression in NK cells in LCL and DCL patients

In order to clarify whether the reduced cytokine production and TLR2 expression in DCL patients was related to reduced gene expression, real-time PCR was done in NK cells of both groups of patients. The gene expression of TLR2, IFN-γ and TNF-α was analyzed in non-stimulated and LPG-stimulated NK cells of 6 LCL and 3 DCL patients as well as 5 healthy controls. DCL vs LCL patients were compared using 2^-ΔΔ*C*T^ method and as a calibrator, LCL 2^−ΔΔ*C*T^ was used. We also compared DCL or LCL vs healthy controls, using controls as calibrators for both studies. The results show a significant up-regulation of IFN-γ gene expression in NK cells of LCL patients after stimulation with LPG, as compared to healthy controls (Table 1, lane 7). In contrast, DCL patients showed a significant down-regulation in IFN-γ gene expression both in non-stimulated NK cells (Table 1, lane 6) as well as after LPG-stimulation (Table 1, lane 9), when compared with LCL patients.

**Table 1:** Transcript expression by quantitative real time PCR in non-stimulated (NS) and LPG-stimulated NK cells (S).

Gene	1	2	3	4	5	6	7	8	9
	Fold change								
TLR2	0.96	1.06	1.06	0.53	0.09	0.18	0.57	0.10	0.18
IFN-γ	1.04	1.57	1.13	2.80	1.00	0.36*	4.08*	1.09	0.26*
TNF-α	1.00	1.34	2.00	1.05	0.62	0.57	1.34	1.20	0.89

TLR2, IFN-γ and TNF-α gene expression in NK cells of healthy controls (n = 5), LCL (n = 6) and DCL (n = 3) patients.

**1**. C_S vs C_NS; **2**. LCL_S vs LCL_NS; **3**. DCL_S vs DCL_NS; **4**. LCL_NS vs C_NS; **5**. DCL_NS vs C_NS; **6**. DCL_NS vs LCL_NS; **7**. LCL_S vs C_S; **8**. DCL_S vs C_S; **9**. DCL_S vs LCL_S. C: healthy controls, LCL: patients with localized cutaneous leishmaniasis, DCL: patients with diffuse cutaneous leishmaniasis. *p<0.05 significant differences.

DCL patients also showed down-regulation of TLR2 gene expression in non-stimulated as well as in LPG-stimulated NK cells, when compared to LCL patients (Table 1, lane 6 and lane 9) or to healthy controls (Table1, lane 5 and lane 8), albeit these differences were not statistically significant. The same holds true for the expression of TNF-α genes, which also showed a non-significant down-regulation in DCL patients, as compared to LCL patients, both in non-stimulated as in LPG-stimulated NK cells (Table 1, lane 6 and lane 9).

Thus, we were able to show that the reduced protein expression of IFN-γ in NK cells of DCL patients correlated with the down-regulation of its gene expression.

## Discussion

The cause of uncontrolled parasite dissemination in DCL patients infected with *Leishmania mexicana* remains an enigma. Although much insight has been gained on the importance of a Th1 response for parasite control in mouse models [14], these data cannot be extrapolated to the human disease. The molecules and mechanisms of innate and adaptive immunities, particularly the role of inflammation, need to be further assessed in the physiopathology of human leishmaniasis. One of the cells that possibly play a role in defining disease severity is the NK cell, since this innate cell is able to produce IFN-γ and TNF-α, both of which are required to activate the leishmanicidal machinery within macrophages. We had previously shown that *Leishmania* LPG is a ligand for TLR2 leading to IFN-γ and TNF-α production [3]. Our current results show that in addition to TLR2, TLR1 and TLR6 are also present in the binding of LPG.

Most studies of TLRs in leishmaniasis have been done in the murine models, where expression of TLR2, TLR4, TLR7, TLR8 and TLR9 have been analyzed and related to disease outcome, together with other contributing factors such as *Leishmania* species and genetic background. Yet data on the role of TLRs obtained from experimental murine leishmaniasis remain controversial: on one hand, enhanced the expression of these TLRs have been related to protection mediated by cytokine production, whereas their absence has been associated with a Th2 response and elevated *Leishmania* numbers [7], [10], [17]–[20]. Contrasting results have shown that the absence of TLR2 during the initial stages of the disease lead to reduced parasite burdens in *L. amazonensis* infected mice, which was associated with more organized granuloma formations [21]. This was supported by data of TLR2^−/−^ mice that achieved a better elimination of the parasite due to reduced inflammatory infiltrates [22].

Yet the role of TLRs in human leishmaniasis has not been thoroughly explored. Some studies have shown that patients with leishmaniasis increase their TLR (1, 2, 3, 4 and 9) expression, as compared to healthy or cured subjects [23]–[29]. Furthermore, TLR expression has been shown to decrease during chronic diseases and with age [29]–[32]. The expression of TLRs also depends on the cell type [23], [30] species/strains and virulence of *Leishmania* parasites [22], [33] or TLR polymorphisms [34].

Little is known of TLRs and NK cell activity in patients with different clinical forms of cutaneous leishmaniasis and to what degree these can be related. Previous studies of NK cells in DCL patients have reported a reduction in NK cell numbers that could be restored after treatment and parasite reduction [1], [35], suggesting that the parasite is able to regulate NK cells. Lieke et al. demonstrated that NK cells incubated with *L. major or L. aethiopica* could lead to death not only of the parasite, but of the NK cell as well [36]. Yet functional modulation of NK cells by this parasite remained to be analyzed. We were therefore interested in analyzing the expression of TLR2, as well as cytokine production by NK cells of patients with LCL and DCL and to evaluate their possible association with disease severity. We were furthermore interested whether specific heterodimers form between TLR2 and TLR1 or TLR6 when binding to LPG and if there is a preferential expression of any of these TLRs in patients with LCL and DCL that could be related to disease severity. We found striking differences in the NK-cell numbers, in the magnitude of TLR expression as well as in IFN-γ and TNF-α productions by NK cells of DCL and LCL patients, which correlated with disease severity: DCL patients showed reduced NK cell numbers (possibly due to NK-cell death), down-regulated TLR2, TLR1 and TLR6 expression as well as reduced cytokine production, as compared to LCL patients. In contrast, LCL patients showed enhanced expression of these TLRs, which correlated with augmented IFN-γ and TNF-α production by their blood NK cells, in addition to enhanced tissue NK cells with IFN-γ and TNF-α staining.

Although tissue lesions of DCL patients showed reduced NK cells with TNF-α staining, they harbored larger NK-negative cells, which stained for TNF-α. Our study did not clarify the nature of these cells, yet due to their size/form, we are tempted to speculate that they could be mast cells, capable of storing preformed TNF-α, that is released upon various stimuli. The elevated amounts of cells containing TNF-α in lesions of DCL patients, possibly account for the intense inflammation and associated tissue damage found in these patients [37]. The tissue damage due to intense inflammation has also been shown in patients with mucocutaneous leishmaniasis [38], [39].

A further finding was that the NK cells of LCL patients expressing TLRs were found within granulomas. Highly organized granuloma structures have been related to host resistance during hepatic leishmaniasis of patients with visceral leishmaniasis caused by *Leishmania donovani* [40]. Within the protective microenvironment created by granuloma formations, NK cells possibly contribute to anti-leishmanial mechanisms by secreting IFN-γ and TNF-α, leading to macrophage activation and helping to create a protective Th-1 environment together with CD4+ cells, both of which are significantly enhanced in LCL, as compared to DCL patients [1], [41]. Our data on TLRs in granulomas in patients with cutaneous leishmaniasis are in accordance with the literature, where TLR9 and TLR2 have been reported [21], [24]. In contrast to the enhanced numbers of NK cells within granulomas found in tissue lesions of LCL patients, DCL lesions show reduced numbers of NK cells scattered within lesions showing no cellular organization, both of which possibly contribute to lack of disease control in these patients.

The enhanced IFN-γ and TNF-α production observed in LCL patients corresponded to females, which showed a more pronounced increase of their IFN-γ production upon prolonged disease evolution (≥4 months). We propose that our results possibly give a new insight into the mechanisms involved in conferring a higher resistance to female patients towards *Leishmania* infections, as has been reported in the literature [37], [42]. Our observations on the lower IFN-γ production in female LCL patients during the first 3 months after infection and the subsequent increase of these cytokine as disease progresses has also been reported by other groups [43]–[46]. IFN-γ production seems to depend on the patient's background, clinical form, age and gender [47]. We hypothesize that this continuous increase in cytokine production in female patients is possibly due to enhanced IFN-γ receptors in their NK cells that ensure autocrine activation and further cytokine production.

In accordance, down-regulation of TLR1, TLR2 and TLR6 expression on NK cells of DCL patients and their diminished IFN-γ and TNF-α cytokine production possibly contributes to disease progression due to their inability to induce leishmanicidal mechanisms of macrophages. The significantly reduced IFN-γ gene expression, and to a lesser extent those of TLR2 and TNF-α, in NK cells of DCL patients, indicates that *Leishmania mexicana* possibly modulates the host immune response through epigenetic mechanisms. This modulation seems not only related to NK cells since reduced levels of IFN-γ and TNF-α have also been reported in other cells of DCL patients [48]–[50]. It is not clear whether the phenotypical and functional modifications of NK cells are the consequence or the cause of *Leishmania* infections, yet it is noteworthy that these patients are not prone to other microbial infections. We are tempted to propose that *Leishmania mexicana* is capable of an epigenetic modulation of the host's transcriptional program, reducing TLR2 expression and cytokine production. If this were the case, the molecular basis of this epigenetic variation remains to be analyzed. The analysis of genes and proteins of the TLR2 signal transduction pathway in NK cells of DCL patients could also add further insight into the cause of disease progression of these patients. Additionally, an analysis of HLA and other susceptibility genes are warranted in DCL patients to shed further light into their enhanced disease severity.

In conclusion, we here present novel information on NK-cell functions in human leishmaniasis. To the best of our knowledge, this is the first report that not only shows the complete activation pathway of LPG binding to TLR2 in NK cells, but also shows that TLR2 can bind to either TLR1 or TLR6. Furthermore our comparative results on NK-cell numbers, phenotypical characteristics and cytokine production in LCL and DCL patients possibly shed new light into the physiopathological mechanisms of the disease. Disease susceptibility of DCL patients is possibly linked to reduced NK cell numbers and reduced activity based on diminished TLR2, TLR1 and TLR6 expression, which in turn reduces their ability to detect *Leishmania* LPG, thus rendering them unable to secrete IFN-γ and TNF-α that are needed to induce leishmanicidal effector mechanisms within phagocytic cells. The phenotypic and functional alterations in NK cells are most probably not the sole cause responsible of parasite dissemination in DCL patients, yet their contribution cannot be ruled out. It remains to be established whether expression of these TLRs are also modified in other cells of DCL patients in order to more clearly establish the role of NK cells in disease susceptibility during *Leishmania mexicana* infections.

### Accession Numbers

Accession links for numbers/ID numbers for genes and proteins mentioned in the text:

TLR1

Gene: http://www.ncbi.nlm.nih.gov/gene/7096

Protein: http://www.uniprot.org/uniprot/Q15399

TLR2

Gene: http://www.ncbi.nlm.nih.gov/gene/7097

Protein: http://www.uniprot.org/uniprot/O60603

TLR6

Gene: http://www.ncbi.nlm.nih.gov/gene/10333

Protein: http://www.uniprot.org/uniprot/Q9Y2C9

CD16

Gene: http://www.ncbi.nlm.nih.gov/gene/2214

Protein: http://www.uniprot.org/uniprot/Q9UPY7

CD56

Gene: http://www.ncbi.nlm.nih.gov/gene/4684

Protein: http://www.uniprot.org/uniprot/P13591

MyD88

Gene: http://www.ncbi.nlm.nih.gov/gene/4615

Protein: http://www.uniprot.org/uniprot/Q99836

IRAK1

Gene: http://www.ncbi.nlm.nih.gov/gene/3654

Protein: http://www.uniprot.org/uniprot/P51617

TRAF6

Gene: http://www.ncbi.nlm.nih.gov/gene/7189

Protein: http://www.uniprot.org/uniprot/Q9Y4K3

IKK-α

Gene: http://www.ncbi.nlm.nih.gov/gene/1147

Protein: http://www.uniprot.org/uniprot/O15111

IKK-β

Gene: http://www.ncbi.nlm.nih.gov/gene/3551

Protein: http://www.uniprot.org/uniprot/O14920

IKK-γ

Gene: http://www.ncbi.nlm.nih.gov/gene/8517

Protein: http://www.uniprot.org/uniprot/Q9Y6K9

IκB-α

Gene: http://www.ncbi.nlm.nih.gov/gene/4792

Protein: http://www.uniprot.org/uniprot/P25963

NF-κB p50

Gene: http://www.ncbi.nlm.nih.gov/gene/4790

Protein: http://www.uniprot.org/uniprot/P19838

NF-κB p65

Gene: http://www.ncbi.nlm.nih.gov/gene/5970

Protein: http://www.uniprot.org/uniprot/Q04206

TNF-α

Gene: http://www.ncbi.nlm.nih.gov/gene/7124

Protein: http://www.uniprot.org/uniprot/Q9UBM5

IFN- γ

Gene: http://www.ncbi.nlm.nih.gov/gene/3458

Protein: http://www.uniprot.org/uniprot/P01579

## Supporting Information

Figure S1 **Expression of TLR1, TLR2 and TLR6 in NK cells stimulated with different TLR2 ligands (LPG, PGN and Pam_3_Cys).** Cell surface expression is indicated by the geometric mean of fluorescence intensity (MIF). These results are the mean ±SEM. *p≤0.05 was considered statistically significant.
(TIFF)
