# Supplementary figures and images for: NK Cell Activity Differs between Patients with Localized and Diffuse Cutaneous Leishmaniasis Infected with Leishmania mexicana: A Comparative Study of TLRs and Cytokines
Source: PLoS One. 2014 Nov 14;9(11):e112410. doi: 10.1371/journal.pone.0112410 (PMC4232367; doi:10.1371/journal.pone.0112410)

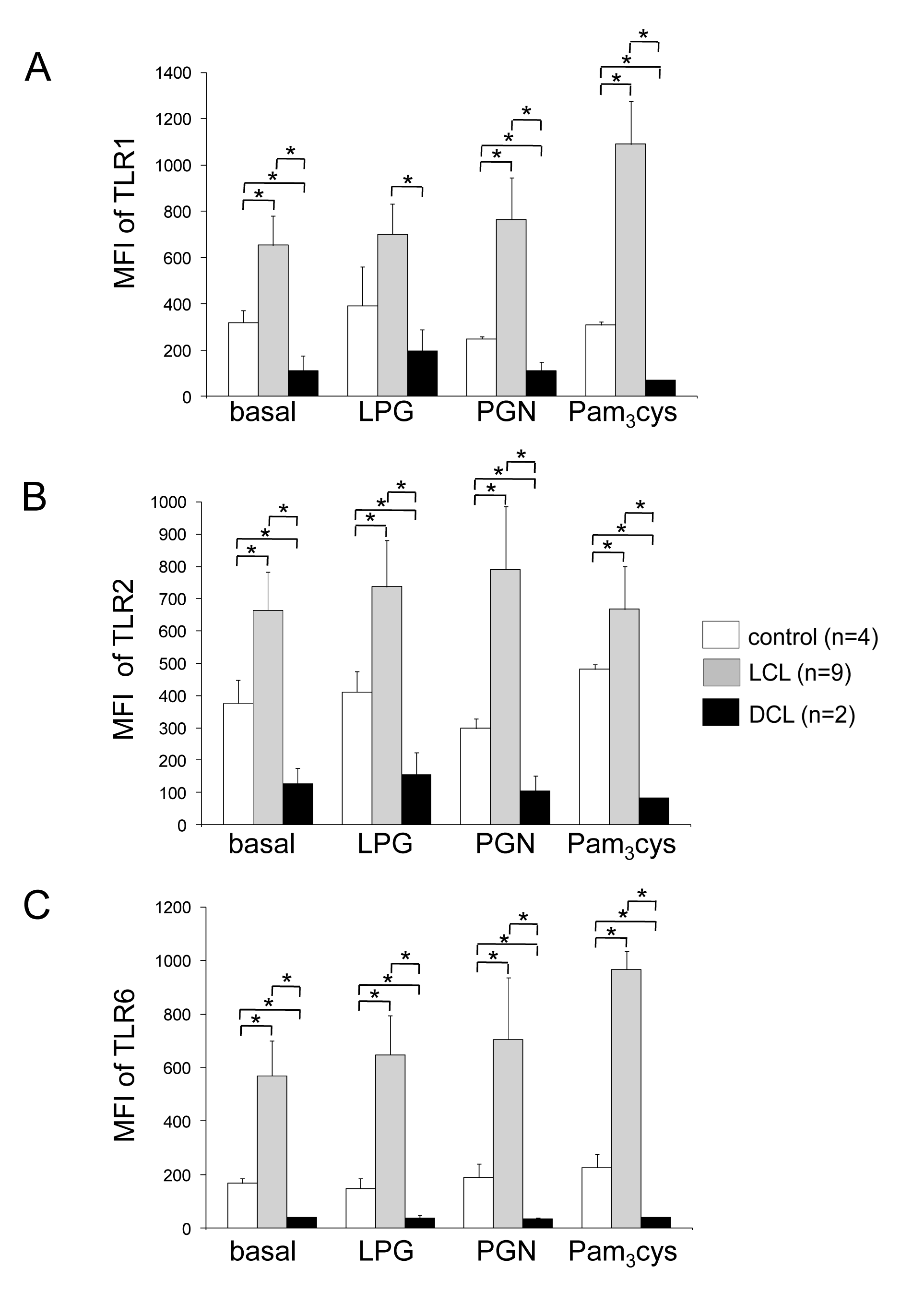

Supplement: Figure S1 — Expression of TLR1, TLR2 and TLR6 in NK cells stimulated with different TLR2 ligands (LPG, PGN and Pam3Cys). Cell surface expression is indicated by the geometric mean of fluorescence intensity (MIF). These results are the mean ±SEM. *p≤0.05 was considered statistically significant. (TIFF) [file pone.0112410.s001.tiff]
